# Vaccination Lies

**Published:** 1897-09

**Authors:** 


### VACCINATION LIES.

Dr. Corfield (as reported in the newspapers) stated at St. George's Hall that the French soldiers practice vaccination and revaccination, and that consequently, although epidemics of small-pox are frequent in Paris, there has been no case of it in the Parisian Guard for seventy years. But upon inquiry from a French army surgeon it turned out:

(1) That small-pox epidemics are not frequent in Paris.

(2) That although every French soldier is vaccinated and revaccinated the mortality from small-pox in the French army during the epidemic of 1871 and 1872 was terrible.

(3) That there is no such corps as the "Parisian Guard." —*Fashions of the Day in Medicine and Science, by H. Strickland Constable. Leng & Co., Kingston-upon-Hull, 1879, page 23.*

